# Natural Polyphenol Corilagin Enhances Osteogenesis and Chondrogenesis Differentiation of Mesenchymal Stem Cells: Implications for Bone and Cartilage Regeneration

**DOI:** 10.3390/molecules31010194

**Published:** 2026-01-05

**Authors:** Thitianan Kulsirirat, Sittisak Honsawek, Mariko Takeda-Morishita, Korbtham Sathirakul

**Affiliations:** 1 Department of Biopharmacy, Faculty of Pharmacy, Srinakharinwirot University, Nakhon Nayok 26120, Thailand; thitianan@g.swu.ac.th; 2 Center of Excellence in Osteoarthritis and Musculoskeleton, Department of Biochemistry, Faculty of Medicine, Chulalongkorn University, King Chulalongkorn Memorial Hospital, Thai Red Cross Society, Bangkok 10330, Thailand; sittisak.h@chula.ac.th; 3 Laboratory of Drug Delivery Systems, Faculty of Pharmaceutical Sciences, Kobe Gakuin University, Kobe 650-8586, Hyogo, Japan; mmtakeda@pharm.kobegakuin.ac.jp; 4 Department of Pharmacy, Faculty of Pharmacy, Mahidol University, Bangkok 10400, Thailand

**Keywords:** corilagin, medicinal plants, mesenchymal stem cells, biomarkers, biomedical fields, regenerative medicine

## Abstract

Corilagin is a hydrolyzable ellagitannin and naturally occurring polyphenolic compound widely distributed in medicinal plants. It is also present in longan (*Dimocarpus longan*), known as *lumyai* in Thailand, a subtropical fruit extensively cultivated across China and Southeast Asia. Corilagin has been reported to exhibit strong antioxidant, anti-inflammatory, hepatoprotective, and anticancer activities through modulation of multiple cellular signaling pathways. However, despite these well-established pharmacological properties, its potential role in regulating bone marrow mesenchymal stem cell (BM-MSC) differentiation has not been fully explored in biomedical applications. In this study, we investigated the effects of corilagin on BM-MSC viability, protein-binding interactions, and lineage-specific differentiation toward osteogenic and chondrogenic pathways. Cytotoxicity assessment using human synovial SW-982 cells demonstrated that corilagin maintained cell viability at concentrations ranging from 1.56 to 50 µg/mL within 48 h, whereas prolonged exposure resulted in a time-dependent reduction in viability. In BM-MSCs, corilagin significantly enhanced osteogenic and chondrogenic differentiation in a dose-dependent manner, as evidenced by increased mineral deposition and cartilage matrix formation, as revealed by Alizarin Red S, Toluidine Blue, and Alcian Blue staining. Quantitative analyses further showed the upregulation of key lineage-specific genes, including Runx2 and osteopontin (OPN) for osteogenesis and Sox9 and aggrecan for chondrogenesis. Protein-binding assays confirmed the molecular interaction capacity of corilagin, supporting its biological activity. Overall, these findings demonstrate that corilagin promotes MSC-mediated osteogenic and chondrogenic differentiation while maintaining acceptable cytocompatibility, highlighting its potential as a natural small-molecule candidate for bone and cartilage tissue engineering and other biomedical fields with regenerative medicine applications.

## 1. Introduction

Bone and cartilage degeneration represent major clinical challenges worldwide, particularly in aging societies where musculoskeletal disorders such as osteoarthritis, osteoporosis, and cartilage injuries are increasingly prevalent [1]. These conditions significantly impair mobility and quality of life and impose a substantial socioeconomic burden. Current clinical treatments including pharmacological interventions, surgical procedures, and joint replacement primarily focus on symptom management rather than true tissue regeneration and are often associated with limited long-term efficacy and adverse side effects [2,3]. Consequently, there is a growing demand for regenerative strategies that can restore bone and cartilage structure and function.

Natural polyphenols constitute a diverse class of plant-derived compounds recognized for their antioxidant, anti-inflammatory, and cytoprotective properties. Several polyphenols, including curcumin, resveratrol, and quercetin, have been extensively studied for their roles in bone and cartilage regeneration. Quercetin has been shown to induce osteoblast (OB) differentiation through activation of Smad signaling and p38 MAPK pathways, while inhibiting the Wnt/β-catenin pathway. In addition, quercetin suppresses RANKL-induced osteoclastogenesis via inhibition of NF-κB activation [4,5,6]. Curcumin has been reported to inhibit osteoclastogenesis by suppressing HMGB1 release through a p38 MAPK–dependent mechanism [7], and previous work from our group demonstrated that curcumin inhibits RANKL-induced osteoclastogenesis [8]. Furthermore, curcumin enhances osteoblast differentiation of human adipose-derived mesenchymal stem cells (MSCs) by inhibiting Wnt/β-catenin signaling, suggesting its regulatory role in bone metabolism [9]. Resveratrol has also been shown to promote osteoblast differentiation through regulation of RUNX2 [10]. In vivo studies further demonstrated that resveratrol could attenuate methotrexate-induced bone loss [11].

MSCs are multipotent stromal cells capable of differentiating into several mesenchymal lineages, including osteoblasts and chondrocytes, making them a cornerstone of regenerative medicine. Enhancing MSC differentiation efficiency is a key strategy for improving outcomes in bone and cartilage tissue engineering. A few natural polyphenols, including resveratrol, curcumin, quercetin, and epigallocatechin gallate (EGCG), have been shown to promote MSC osteogenesis and chondrogenesis by modulating cellular redox status, mitochondrial activity, and downstream transcriptional regulators such as Runx2, osteopontin (OPN), Sox9, and Aggrecan [12,13]. Given that corilagin shares many biochemical properties with these differentiation-enhancing polyphenols, it is reasonable to hypothesize that it may exert similar regulatory activity on MSC lineage commitment. Nevertheless, its specific role in directing osteogenic and chondrogenic pathways has not been systematically explored.

**Figure 1:**
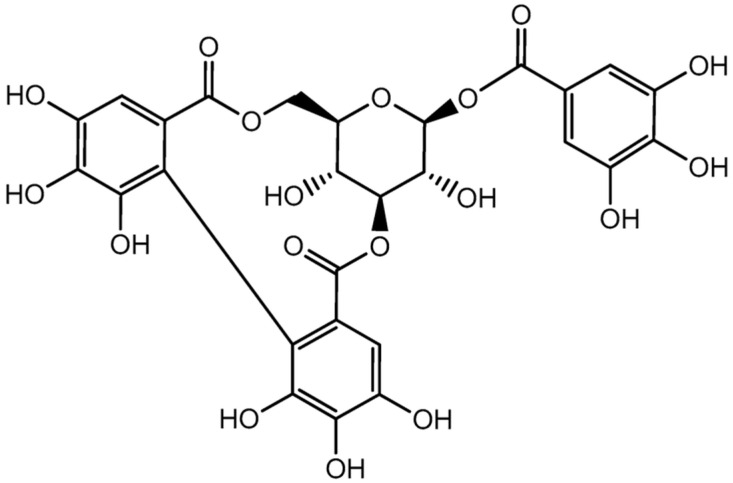
The chemical structure of corilagin.

Corilagin (Figure 1) [14] is an ellagitannin and naturally occurring polyphenolic compound found in a wide range of medicinal plants [15]. It has been isolated from numerous species, including *Euphoria longana* (longan) [16], *Dimocarpus longan* [17], *Punica granatum* [18], *Terminalia chebula* [19], *Terminalia catappa* [20], *Rosa rugosa* and *Eugenia caryophyllata* [21], *Phyllanthus niruri* [22,23], *Phyllanthus amarus* [24], *Emblica officinalis* [25], *Syzygium cumini* [26], and *Lumnitzera racemosa* [27]. Over the past decade, corilagin has attracted considerable scientific interest due to its diverse pharmacological activities. Numerous studies have reported that corilagin exhibits strong antioxidant [28,29], anti-inflammatory [30], hepatoprotective [31], antimicrobial [32], antihypertensive [33], antiatherogenic [34], antidiabetic [35,36], and anticancer properties [37]. These biological effects are largely attributed to its ability to regulate oxidative stress and modulate key signaling pathways, including NF-κB, MAPK, PI3K/Akt, and TGF-β signaling cascades [38,39].

In addition, joint-related disorders such as osteoarthritis involve complex interactions among bones, cartilage, and synovial tissues. Synovial fibroblast-like cells play a critical role in joint homeostasis and inflammatory responses, yet limited information is available regarding the cytotoxic and time-dependent effects of corilagin on synovial cells. Understanding these effects is essential for evaluating the translational feasibility and safety profile of corilagin in joint-related regenerative applications.

In this study, we aimed to investigate the effects of corilagin on cell viability, osteogenic differentiation, and chondrogenic differentiation of bone marrow-derived mesenchymal stem cells (BM-MSCs), as well as its cytotoxic profile in synovial SW-982 cells. By integrating cytotoxicity assessment, histological staining, and gene expression analysis, this work is designed to clarify the biological activity of corilagin in bone and cartilage regeneration and to address current gaps in knowledge regarding its role as a bioactive polyphenol for musculoskeletal tissue regeneration.

## 2. Results

### 2.1. Culture Expansion of BM-MSCs

**Figure 2:**
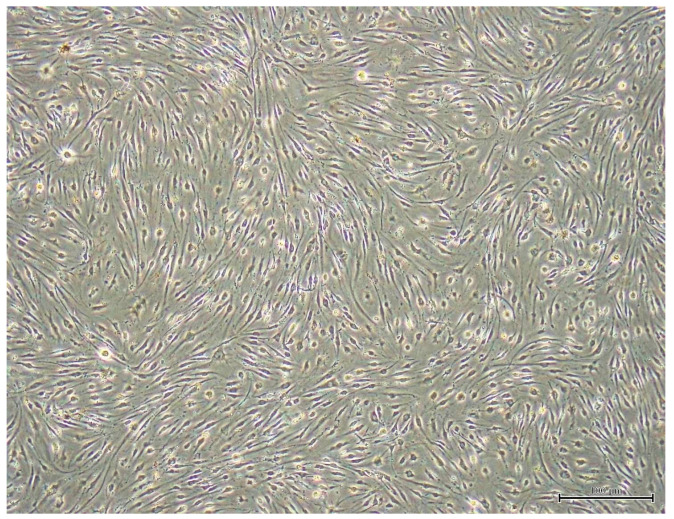
Representative cell morphology of BM-MSCs at the third passage as observed under a light microscope at 4× magnification.

BM-MSCs cryopreserved were thawed at 37 °C in a water bath and revived using pre-warmed culture medium. The cells were then centrifuged at 1000 rpm for 5 min. The resulting pellet was gently resuspended in DMEM-HG and seeded into T-75 flasks (Corning, NY, USA). After three days of incubation, the cells had spread and adhered to the plastic surface of the culture flasks. By 4–5 days, the cells displayed a homogeneous spindle-shaped, fibroblast-like morphology. Over time, the cells proliferated rapidly and formed clusters. Cultures reached 80–90% confluence within 7–10 days. After the first subculturing, each subsequent passage required approximately 4–5 days. The cells maintained a homogeneous morphology throughout the culture period until termination (Figure 2).

### 2.2. Effect of Corilagin on the Cell Viability of Differentiating BM-MSCs

**Figure 3:**
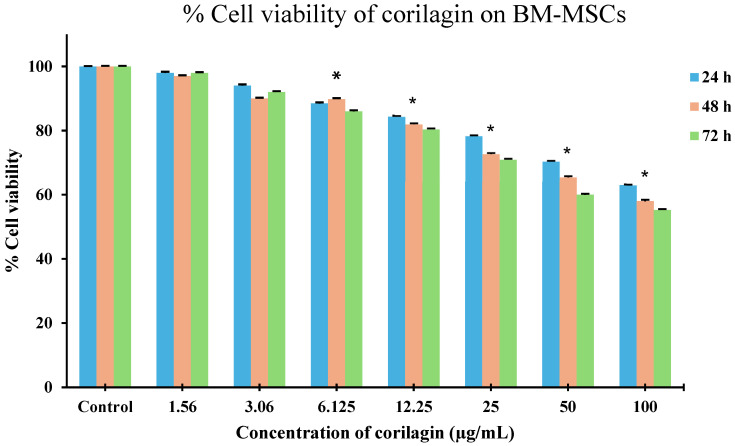
Effects of corilagin on BM-MSC viability assessed by MTT assay at 24, 48, and 72 h. Cells were treated with increasing concentrations of corilagin (1.56–100 µg/mL). Data are presented as mean ± SD (n = 3). Statistical analysis was performed using one-way ANOVA followed by Tukey’s post hoc test. *p* < 0.05 vs. control; * *p* < 0.05 vs. 6.125 µg/mL.

To investigate the effect of corilagin on cell viability, an MTT assay was performed to assess mitochondrial reducing activity. As shown in Figure 3, the optical density (OD) values of BM-MSCs remained comparable to the control at 24 h across concentrations ranging from 1.56 to 12.5 µg/mL, indicating no apparent cytotoxic effect at this time point. At 48 and 72 h, cell viability at lower concentrations (1.56–3.06 µg/mL) remained similar to the control, whereas a modest reduction in viability was observed at 12.5 µg/mL, suggesting a time- and dose-dependent effect at higher concentrations. In contrast, corilagin significantly decreased cell viability at concentrations above 12.5 µg/mL at all tested time points (24, 48, and 72 h), indicating that higher concentrations exert cytotoxic effects on BM-MSCs. Together, these results suggest that corilagin is well tolerated at lower concentrations, while prolonged exposure to higher concentrations may inhibit BM-MSC proliferation.

### 2.3. In Vitro Osteogenic and Chondrogenic Differentiation with Histological Analysis of Chondrogenic Differentiation

**Figure 4:**
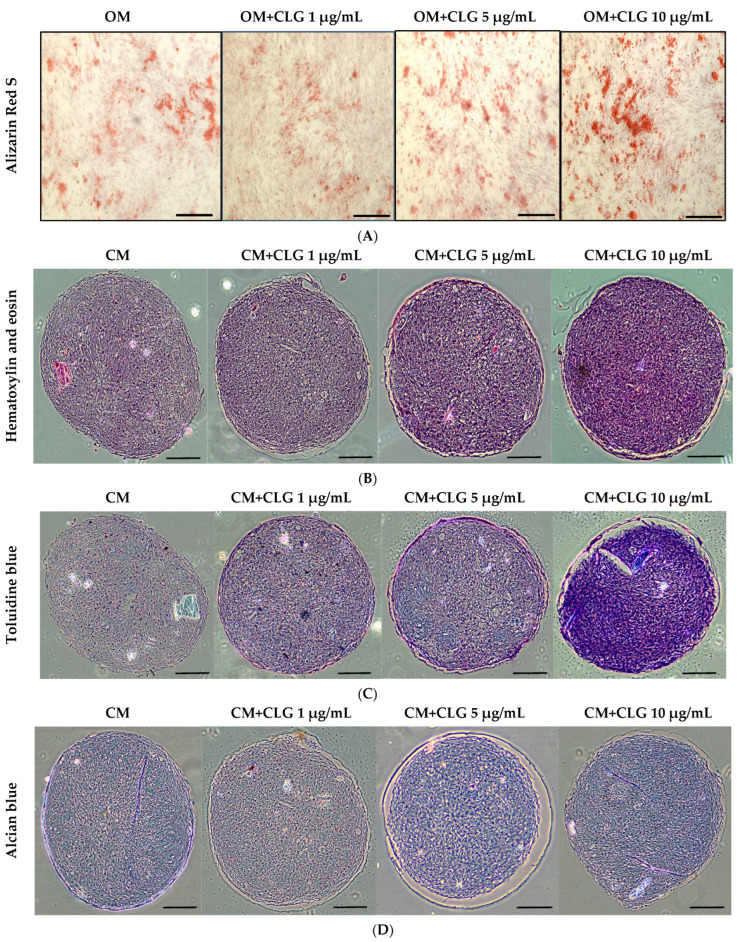
(**A**) Alizarin red S staining revealed the presence of calcium deposits 21 days after the induction of osteogenic medium (OM) in the presence of CLG at varying concentrations (1 μg/mL, 5 μg/mL, and 10 μg/mL). The scale bar (⎯) represents 200 µm. (**B**) Pellet cultures of BM-MSCs were sectioned and stained with H&E, showing the morphology at 21 days after the induction of chondrogenic medium (CM) with CLG at varying concentrations (1 μg/mL, 5 μg/mL, and 10 μg/mL). The scale bar (⎯) represents 200 µm. (**C**) Pellet cultures of BM-MSCs were sectioned and stained with toluidine blue, showing glycosaminoglycans at 21 days after the induction of chondrogenic medium (CM) with CLG at varying concentrations (1 μg/mL, 5 μg/mL, and 10 μg/mL). The scale bar (⎯) represents 200 µm. (**D**) Pellet cultures of BM-MSCs were sectioned and stained with Alcian blue, showing proteoglycans at 21 days after the induction of chondrogenic medium (CM) with CLG at varying concentrations (1 μg/mL, 5 μg/mL, and 10 μg/mL). The scale bar (⎯) represents 200 µm.

Third-passage BM-MSC cultures were subjected to in vitro differentiation assays to evaluate their mesenchymal multipotent potential. Morphological changes were observed, with cells adopting a spindle-shaped appearance. As shown in Figure 4, the differentiation potential was confirmed by positive osteogenic and chondrogenic staining.

**Figure 5:**
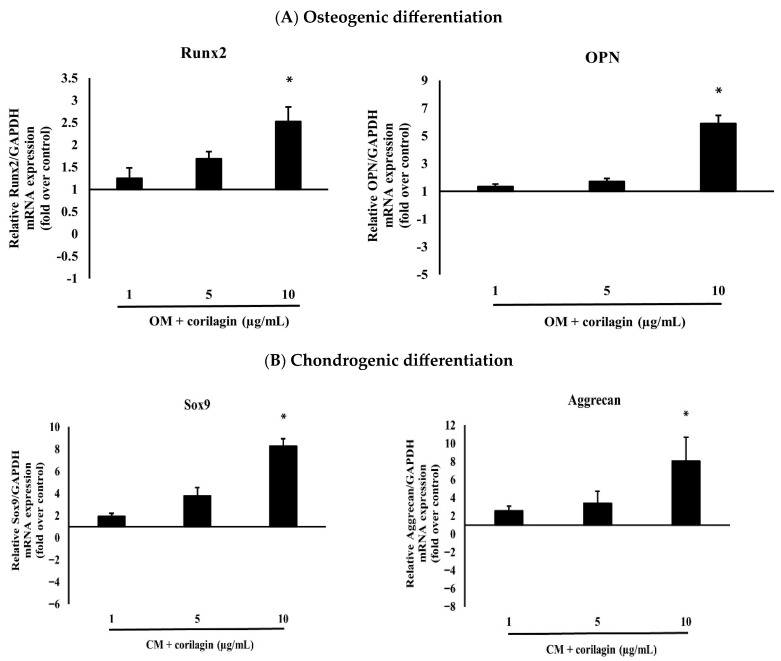
(**A**) The effects of different doses of corilagin (1, 5 and 10 µg/mL) on osteogenic differentiation potentials (Runx2, OPN) of BM-MSCs on day 21. Gene expression of differentiation potentials was assessed using real-time PCR. The bar means SD. Data is shown as mean ± SD. Statistical significance was calculated using a one-way ANOVA test and significance is represented on graphs as * *p*-value < 0.05. (**B**) The effects of different doses of corilagin (1, 5 and 10 µg/mL) on chondrogenic differentiation potentials (Sox9, Aggrecan) of BM-MSCs on day 21. Gene expression of differentiation potentials was assessed using real-time PCR. The bar means SD. Data is shown as mean ± SD. Statistical significance was calculated using a one-way ANOVA test and significance is represented on graphs as * *p*-value < 0.05.

For osteogenic induction, cells were seeded at a density of 5 × 10^4^ cells per well in six-well plates (triplicates) and cultured in growth medium. After 24 h, when the cells reached approximately 90% confluency, BM-MSCs were treated with osteogenic induction medium (OM) supplemented with corilagin at different concentrations (1, 5, and 10 μg/mL). Cells cultured in OM without corilagin served as controls. After 21 days, mineralized matrix deposition was confirmed by Alizarin Red S staining (Figure 4A). In addition, osteogenic potency was validated by examining the mRNA expression of osteogenic-specific markers, including Runt-related transcription factor 2 (Runx2) and Osteopontin (OPN) (Figure 5A). Treatment with corilagin markedly upregulated the expression of these markers in a dose-dependent manner compared with the control group.

For chondrogenic induction, BM-MSCs were further analyzed to evaluate their chondrogenic differentiation capacity using a pellet culture system. Pellets were cultured in chondrogenic medium (CM) supplemented with corilagin at different concentrations (1, 5, and 10 μg/mL) or in CM containing 1% FBS as a control. The cells aggregated and formed dense spheroidal pellets, which were harvested after 21 days for histological analysis. Staining with Hematoxylin & Eosin (H&E) revealed pellet morphology (Figure 4B), toluidine blue confirmed the presence of glycosaminoglycans (Figure 4C), and Alcian blue demonstrated enrichment of sulfated proteoglycans (Figure 4D). In addition, the mRNA expression of chondrogenic-specific markers, including SRY-related transcription factor 9 (Sox9) and Aggrecan, was assessed (Figure 5B). Pellets treated with corilagin at all tested concentrations, particularly at 10 μg/mL, exhibited significantly higher expression levels compared with the CM control group (*p* < 0.05), indicating that corilagin promoted chondrogenic differentiation of BM-MSCs in a dose-dependent manner.

### 2.4. Culture Expansion of Human Synovial Cell Lines (SW-982)

**Figure 6:**
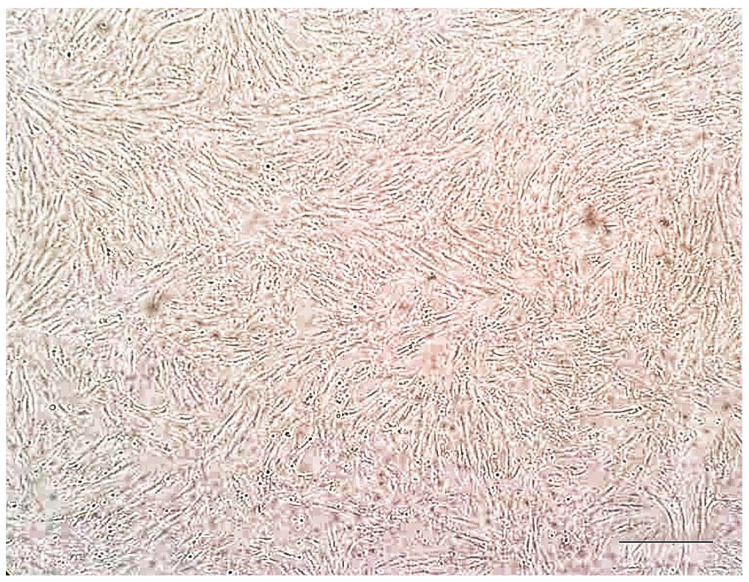
Representative cell morphology of SW-982 cells as observed under a light microscope at 4× magnification. The scale bar (⎯) represents 100 µm.

Cryopreserved SW-982 cells were thawed at 37 °C in a water bath and immediately transferred into pre-warmed culture medium. The cell suspension was centrifuged at 1000 rpm for 5 min, after which the pellet was resuspended in DMEM supplemented with 10% fetal bovine serum (FBS) and 1% penicillin–streptomycin (P/S). The cells were then seeded into T-75 flasks and maintained in a humidified incubator at 37 °C with 5% CO_2_. Within three days, the cells had attached and spread across the culture surface, exhibiting a uniform spindle-shaped, fibroblast-like morphology. As the culture progressed, SW-982 cells proliferated rapidly and began forming dense clusters. As shown in Figure 6, cultures reached approximately 80–90% confluence within 5 days. Following the initial subculture, each subsequent passage required roughly 7 days. Throughout the entire culture period, the cells retained a stable and homogeneous morphology until the experiment was terminated.

### 2.5. Effect of Corilagin on the Cell Viability of Differentiating SW-982 Cells

**Figure 7:**
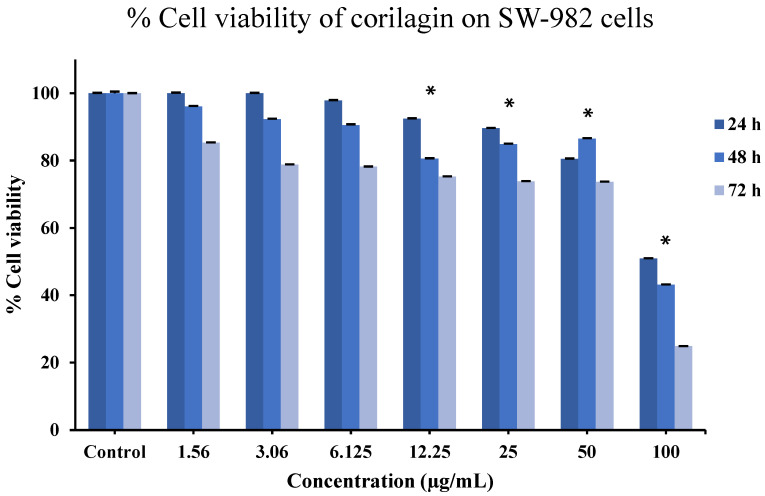
Effects of corilagin on SW-982 cells viability assessed by MTT assay at 24, 48, and 72 h. Cells were treated with increasing concentrations of corilagin (1.56–100 µg/mL). Data are presented as mean ± SD (n = 3). Statistical analysis was performed using one-way ANOVA followed by Tukey’s post hoc test. * *p* < 0.05 vs. control.

To evaluate the effect of corilagin on cell viability, an MTT assay was performed based on mitochondrial metabolic activity. As shown in Figure 7, the viability of SW-982 cells remained comparable to the control at 24 h across concentrations ranging from 1.56 to 50 µg/mL. However, at 48 h, a reduction in cell viability was observed starting at 3.06 µg/mL, indicating the onset of a dose-dependent effect. At 72 h, decreased cell viability was evident even at the lowest tested concentration (1.56 µg/mL), suggesting a pronounced time-dependent cytotoxic effect. These results indicate that while short-term exposure to corilagin is well tolerated by SW-982 cells, prolonged exposure leads to reduced cell viability in both a time- and concentration-dependent manner.

### 2.6. Corilagin Protein Binding Assays in Artificial Synovial Fluid

Equilibrium binding was evaluated after 4 h of incubation. In artificial synovial fluid (ArSF), the protein-binding assay demonstrated that corilagin at concentrations of 1, 5, and 10 mg/mL exhibited progressively higher binding percentages as the concentration increased (Table 1).

## 3. Discussion

Corilagin, an ellagitannin widely recognized in TCM formulations, is one such compound with a documented history of medicinal use. Beyond traditional applications, corilagin is also present in longan (*Dimocarpus longan*), where it is known as lumyai, a subtropical fruit extensively cultivated in China and throughout Southeast Asia, including Thailand, Vietnam, and the Philippines.

Our cytotoxicity data indicate that corilagin is well tolerated by BM-MSCs at concentrations below 12.5 μg/mL and by synovial SW-982 cells up to 50 μg/mL within 48 h. These observations align with previous reports describing the low toxicity of corilagin and related ellagitannins in various mammalian cells [14,15,24,25,26,27] and are consistent with findings that polyphenols often enhance cell viability by reducing oxidative stress and stabilizing mitochondrial function [40,41]. Given that mitochondrial metabolism plays a crucial role in MSCs lineage commitment [42,43], corilagin’s cytoprotective activity may create a cellular environment favorable for differentiation. However, the inhibition of cell proliferation observed at 72 h suggests a time-dependent cytotoxic effect. Prolonged exposure to corilagin may lead to the accumulation of toxic metabolites or induce apoptosis, which could explain the observed decrease in cell viability at this time point. To better understand these time-dependent effects, future studies will focus on investigating the molecular pathways involved in this cytotoxic effect and the long-term consequences of prolonged corilagin exposure on cell viability and differentiation.

A key finding of this study is the dual enhancement of osteogenesis and chondrogenesis in BM-MSCs. Increased mineral deposition and elevated expression of osteogenic genes (Runx2, OPN) confirm the pro-osteogenic action of corilagin, while improved glycosaminoglycan/proteoglycan accumulation and upregulation of Sox9 and Aggrecan substantiate its pro-chondrogenic effect. These outcomes resemble the actions of other polyphenols such as resveratrol, quercetin, and EGCG, which are known to enhance MSC differentiation through redox modulation, epigenetic regulation, and metabolic reprogramming [44,45,46]. Corilagin’s molecular structure, characterized by multiple phenolic hydroxyl groups, may confer similar ROS-scavenging and transcription-modulating properties.

The differentiation enhancing effects observed here may also involve regulation of inflammatory pathways. NF-κB inhibition is a well-established action of corilagin and has been shown to promote osteogenesis by alleviating inflammation-induced suppression of Runx2 [47,48,49]. Likewise, activation of PI3K/Akt signaling enhances both osteogenic and chondrogenic lineage commitment [50]. Some ellagitannins have been shown to activate Akt and ERK pathways during osteogenesis [51], suggesting that corilagin may exert comparable effects. Further mechanistic studies will be needed to verify pathway-specific roles.

The cytotoxicity exposure to corilagin may lead to the accumulation of toxic metabolites or induce apoptosis, which could explain the observed inhibition of cell proliferation at 72 h. Future experiments will be designed to further investigate these mechanisms and determine the underlying causes of cytotoxicity at longer treatment durations.

Protein-binding assays revealed modest but concentration-dependent binding of corilagin in artificial synovial fluid. Although the binding percentages were relatively low, this behavior is consistent with reports that polyphenols form transient interactions with synovial proteins and extracellular matrix components [52]. This approach is particularly useful for joint diseases such as osteoarthritis, as specific interactions may enhance local retention time within the joint while minimizing systemic side effects, an advantageous feature for intra-articular applications.

The implications of these findings are particularly relevant given the increased prevalence of osteoporosis and osteoarthritis in aging populations. Aging is associated with impaired MSC osteogenesis, reduced chondrogenic potential, and elevated oxidative stress, collectively diminishing tissue regeneration capacity [53,54,55]. Natural compounds capable of restoring these functions are therefore of considerable therapeutic interest. The combined osteo-chondrogenic enhancement demonstrated by corilagin suggests potential use as a small-molecule adjunct in tissue engineering constructs, MSCs based therapies, or biomaterial scaffolds.

This study also highlights the potential translational applications of corilagin. Small molecules that promote lineage-specific differentiation are increasingly integrated into bioactive scaffolds, hydrogels, and 3D-printed constructs for bone and cartilage repair [56,57]. The demonstrated ability of corilagin to enhance mineralization and matrix production supports its inclusion in such platforms. Additionally, its moderate synovial fluid protein-binding could be advantageous for joint-targeted delivery.

Despite the promising findings of the present study, several limitations should be acknowledged. First, the molecular mechanisms underlying corilagin’s actions remain undefined and the absence of corilagin-only differentiation controls under osteogenic and chondrogenic conditions. Therefore, Future studies will incorporate corilagin-only treatment groups to further clarify whether corilagin acts as a direct differentiation inducer or primarily as a differentiation enhancer. Second, the current study is limited to in vitro evaluations. In vivo studies are needed to assess pharmacokinetics, systemic distribution, biodegradation, and therapeutic effects in disease models such as osteoporotic bone defects or cartilage injury. Finally, potential synergistic effects between corilagin and biomaterial scaffolds or growth factors should be explored.

## 4. Materials and Methods

Corilagin (C_27_H_22_O_18_, ≥98% purity, Batch no.0545840-4) was purchased from Cayman Chemical (Ann Arbor, MI, USA). Phosphoric acid (H_3_PO_4_) was purchased from Merck (Darmstadt, Germany). Methanol (CH_3_OH, HPLC grade), acetonitrile (ACN, HPLC grade) and dimethyl sulfoxide (DMSO) were purchased from Honeywell Burdick & Jackson (Muskegon, MI, USA). Milli-Q^®^ water (Millipore, Bedford, MA, USA) was used for all studies. All other chemicals were obtained from a commercially analytical grade reagent. MTT reagent (3- (4,5-dimethylthiazol-2-yl)-2,5-diphenyltetrazolium bromide) were purchased from AppliChem GmbH (Damstadt, Germany). Dulbecco’s Modified Eagle Medium-high Glucose (DMEM-HG), 0.25% trypsin-EDTA, fetal bovine serum (FBS), and penicillin–streptomycin (P/S) solution were purchased from Gibco (Waltham, MA, USA).

Primary Bone Marrow-Derived Mesenchymal stem cells from normal human (BM-MSCs, ATCC^®^ number PCS-500-012TM, Lot: 70017526) and human synovial sarcoma cell line (SW-982, HTB-93^TM^, Lot: 63971143) were purchased from American Type Culture Collection (Manassas, VA, USA).

### 4.1. Cell Culture

BM-MSCs and SW-982 cells were cultured in DMEM-HG supplemented with 10% FBS and 1% penicillin/streptomycin. Cell culture was maintained at 37 °C in a 5% CO_2_ incubator. Culture medium was replaced every 3 days. If cells expanded more than 80% of the culture flask, then BM-MSCs, and SW-982 cells were detached with 0.25% Trypsin-EDTA and recultured with complete medium. BM-MSCs were used for all experiments to ensure optimal differentiation capacity. All experiments were performed using at least three independent replicates.

### 4.2. Cytotoxicity Study

The toxicity of corilagin was evaluated in BM-MSCs and SW-982 cells using an MTT assay (AppliChem GmbH, Darmstadt, Germany). BM-MSCs and SW-982 were seeded into 96-well plate at a density of 2 × 10^4^ cells/well and incubated at 37 °C and 5% CO_2_ for 24 h. The old medium was replaced with various concentrations of corilagin (1.56, 3.125, 6.25, 12.5, 25, 50, and 100 μg/mL) dispersed in the culture medium while the control cells were incubated with fresh DMEM and 0.05% (*v*/*v*) DMSO medium. The cultured plates were incubated for 24, 48, and 72 h at 37 °C and 5% CO_2_. Afterwards, the medium was discarded, and the cells in each well were washed with PBS and replaced by 100 μL of 0.5 mg/mL MTT solution. The cells were then incubated for another 3 h at 37 °C under 5% CO_2_, after which MTT solution in each well was carefully discarded and replaced by 50 μL of DMSO to dissolve formazan crystals. Next, the absorbance was measured by a microplate reader (CLARIOstar, BMG LABTECH^®^, Ortenberg, Germany) at a wavelength of 570 nm. The results were illustrated as a percentage of cell viability. The cell viability was calculated relative to the control using the following formula:

(1) $$\%\;Cell\;Viability=\frac{Absorbance\;of\;treated\;cells}{Absorbance\;of\;control\;cells}\;\times \;100$$

### 4.3. Osteogenic and Chondrogenic Differentiation of the BM-MSCs Cells

Osteogenesis: BM-MSCs were seeded at a density of 5000 cells/cm^2^ in freshly prepared osteogenic medium containing 10 nM dexamethasone, 10 mM β-glycerophosphate, and 50 μg/mL ascorbate-2-phosphate, supplemented with 10% fetal bovine serum (FBS). Cultures were maintained in media containing corilagin at concentrations of 1, 5, or 10 μg/mL for 21 days, with medium replaced every 3 days. The concentrations of 1, 5, or 10 µg/mL range were subsequently selected for differentiation experiments as a non-cytotoxic concentration range. For assessment of extracellular calcium deposition, cells were fixed in 100% ethanol and stained with 0.2% (*w*/*v*) Alizarin Red S for 40 min at room temperature. Excess dye was removed by repeated washing with distilled water, and mineralized nodules were examined under an inverted light microscope.

Chondrogenesis: BM-MSCs were cultured to form cell pellets in 15 mL polypropylene tubes (Corning, NY, USA). Approximately 2 × 10^5^ cells were resuspended in freshly prepared chondrogenic medium containing 10 nM dexamethasone (Sigma-Aldrich), 10 ng/mL transforming growth factor-β3 (TGF-β_3_; ProSpec, Rehovot, Israel), and 6.25 μg/mL insulin–transferrin–selenium (ITS supplement; MP Biomedicals, Irvine, CA, USA). Pellets were maintained for 21 days in media supplemented with corilagin at final concentrations of 1, 5, or 10 μg/mL, with medium replaced every 3 days. The concentrations of 1, 5, or 10 µg/mL range were subsequently selected for differentiation experiments as a non-cytotoxic concentration range. For histological analysis, pellets were fixed in formalin, embedded in paraffin, and sectioned at 4–5 μm. Sections were stained with Hematoxylin & Eosin (H&E) to assess morphology, Alcian Blue to detect glycosaminoglycan enrichment, and Toluidine Blue to visualize polysaccharides. Stained sections were examined under an inverted light microscope.

### 4.4. RNA Isolation and Real-Time PCR Analysis

The differences in messenger RNA (mRNA) levels of osteogenic and chondrogenic-related genes in mesenchymal stem cells (BM-MSCs) treated with corilagin at various concentrations (1, 5, and 10 μg/mL) or without treatment (control) were analyzed by quantitative real-time PCR (qRT-PCR). Total RNA was extracted on days 0 and 21 of the induction period using the FavorPrep Blood/Cultured Cell Total RNA Mini Kit (FAVORGEN, Ping-Tung, Taiwan) following the manufacturer’s instructions. qRT-PCR was performed using the SensiFAST™ SYBR^®^ Lo-ROX One-Step Kit on an Mx3005P Real-Time PCR System (Stratagene, Agilent Technologies, Waldbronn, Baden-Württemberg, Germany). Reactions were conducted in a total volume of 20 μL, containing 10 μL of 2× SensiFAST SYBR Green Lo-ROX reaction mix, 0.5 μM of each primer, and 1 μL of cDNA template. The thermal cycling conditions included an initial denaturation at 95 °C for 10 min, followed by 40 cycles of amplification (95 °C for 15 s, 60 °C for 30 s, 72 °C for 30 s), and a final melt curve analysis to assess the specificity of the PCR products. Primer sequences specific to each target gene are listed in Table 2. mRNA expression levels were normalized to the housekeeping gene GAPDH [58], and relative expression was calculated using the ΔΔCT method. The ΔCT values were determined by subtracting the threshold cycle (CT) of the gene of interest from the CT value of GAPDH. The ΔΔCT was then calculated by subtracting the ΔCT of the control group from the ΔCT of the experimental groups. Graphs are presented showing the relative gene expression levels compared to the control group at day 0.

### 4.5. Corilagin Protein Binding Assays in Artificial Synovial Fluid

The protein binding of corilagin was evaluated using rapid equilibrium dialysis (RED) inserts with an 8000 Da molecular weight cut-off in a reusable base plate, following the manufacturer’s instructions. Each assay was conducted in triplicate. Corilagin was tested at concentrations of 1, 5, and 10 μg/mL in artificial synovial fluid (ArSF) formulated to mimic the biochemical environment of human synovial fluid (Hyaluronic acid 3 mg/mL, Albumin 10 mg/mL, γ-globulin 0.5 mg/mL, Phospholipid 0.2 mg/mL). Parallel control assays were performed in phosphate-buffered saline (PBS, pH 7.4). For each assay, 400 μL of corilagin solution was placed in the donor chamber and 600 μL of PBS in the receiver chamber. The assemblies were incubated at 37 °C on a horizontal shaker at 250 rpm, and equilibrium was achieved after 4 h, at which point samples were collected. Protein binding was quantified using a validated high-performance liquid chromatography (HPLC) method, and the binding values were calculated as described

(2) $$\%\;Free=\frac{Concentration\;buffer\;chamber}{Concentration\;sample\;chamber}\times 100$$

(3) % Bound=100%−% Free

### 4.6. Analysis of the Protein Binding Assays of Corilagin by HPLC

**Figure 8:**
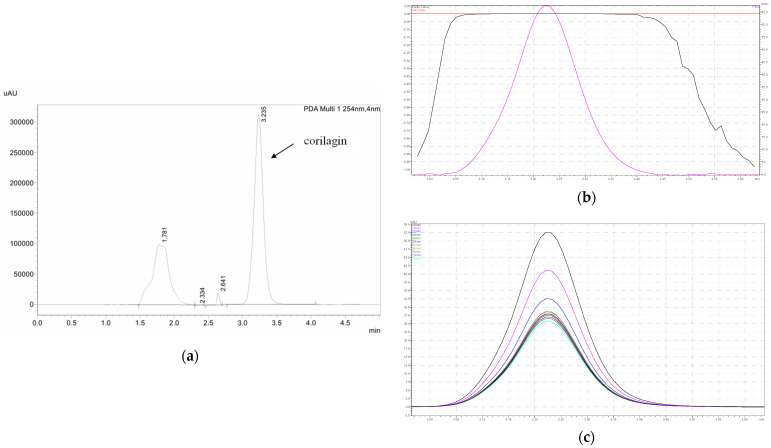
(**a**) HPLC chromatogram of corilagin, (**b**) peak purity of corilagin shown in the pink color, and (**c**) UV spectrum in 254 nm of corilagin shown in the navy blue.

HPLC analysis was performed on a Shimadzu LC-20A (Kyoto, Japan) equipped with photodiode array detector (PDA). A reverse-phase analytical Hypersil^®^ GOLD C18 column (150 mm × 4.6 mm, 0.5 μm particle size, Thermo Scientific, Waltham, MA, USA) was used in this experiment. A mixture of acetonitrile and 0.5% (*v*/*v*) phosphoric acid was used in a 79:21 ratio to make up the mobile phase of the experiment. Flow rate was set at 1 mL/min. The injection volume was 20 μL. Detection wavelength was 254 nm. Prior to use in the content analysis, these HPLC conditions were validated as per the International Committee of Harmonization (ICH) guidelines [59]. The calibration range was 0.10 to 100 μg/mL with R2 of 0.9998. Limits of detection (LOD) and quantification (LOQ) were 0.0450 ± 0.0018 and 0.1400 ± 0.0057 μg/mL, respectively. The HPLC chromatogram, peak purity, and UV spectrum of corilagin are shown in Figure 8.

### 4.7. Statistical Analysis

Statistical analyses were performed using SPSS version 20.0 (IBM Corp, Armonk, NY, USA). Data are presented as mean ± SD (n = 3). Statistical analysis was performed using one-way ANOVA followed by Tukey’s post hoc test. *p* < 0.05 vs. control.

## 5. Conclusions

In this study, we investigated the effects of corilagin, a naturally occurring ellagitannin polyphenol, on mesenchymal stem cell (MSC) viability, molecular interactions, and lineage-specific differentiation toward osteogenic and chondrogenic pathways. Our findings demonstrate that corilagin exhibits acceptable cytocompatibility within a defined concentration range, as evidenced by viability assays in both BM-MSCs and synovial SW-982 cells, while prolonged exposure revealed time- and concentration-dependent effects. In particular, corilagin significantly promoted osteogenic and chondrogenic differentiation of BM-MSCs in a dose-dependent manner. These effects were supported by enhanced mineral deposition and cartilage matrix formation, as well as the upregulation of key lineage-specific markers, including Runx2 and osteopontin for osteogenesis, and Sox9 and aggrecan for chondrogenesis. Its low cytotoxicity to human synovial cells and moderate protein-binding behavior in artificial synovial fluid further suggest that corilagin is well suited for the musculoskeletal microenvironment. Overall, these findings identify corilagin as a promising small-molecule regulator for bone and cartilage tissue engineering. The results also indicate potential for future translational development, supporting the possibility that corilagin may contribute to regenerative strategies for bone and cartilage repair in clinical applications.

## Figures and Tables

**Table 1:** Effect of corilagin concentration on the bound fraction in artificial synovial fluid.

Concentration of Corilagin in ArSF (µg/mL) (N = 3)	Average of % Bound in ArSF
1	1.22 ± 1.07
5	3.37 ± 1.70
10	5.96 ± 3.27

**Table 2:** Primer oligonucleotide sequences used for real-time PCR.

Gene	Forward Primer 5-3	Reverse Primer 5-3
Runx2	5′TATGGCACTTCTTCAGGATCC′3	5′GCGTCAACACCATCATTCTGG′3
OPN	5′TGAAACGAGTCAGCTGGATG′3	5′TGAAATTCATGGCTGTGGAA′3
Sox9	5′ATCTGAAGAAGGAGAGCGAG′3	5′TCAGAAGTCTCCAGAGCTTG′3
Aggrecan	5′TGAGGAGGGCTGGAACAAGTACC′3	5′GGAGGTGGTAATTGCAGGGAACA′3
GAPDH	5′GTGAAGGTCGGAGTCAACGG′3	5′TCAATGAAGGGGTCATTGATGG′3

## Data Availability

The original contributions presented in this study are included in the article. Further inquiries can be directed to the corresponding author.
