# Correction: Kulsirirat et al. Natural Polyphenol Corilagin Enhances Osteogenesis and Chondrogenesis Differentiation of Mesenchymal Stem Cells: Implications for Bone and Cartilage Regeneration. *Molecules* 2026, *31*, 194

**DOI:** 10.3390/molecules31030412

**Published:** 2026-01-26

**Authors:** Thitianan Kulsirirat, Sittisak Honsawek, Mariko Takeda-Morishita, Korbtham Sathirakul

**Affiliations:** 1 Department of Biopharmacy, Faculty of Pharmacy, Srinakharinwirot University, Nakhon Nayok 26120, Thailand; thitianan@g.swu.ac.th; 2 Center of Excellence in Osteoarthritis and Musculoskeleton, Department of Biochemistry, Faculty of Medicine, Chulalongkorn University, King Chulalongkorn Memorial Hospital, Thai Red Cross Society, Bangkok 10330, Thailand; sittisak.h@chula.ac.th; 3 Laboratory of Drug Delivery Systems, Faculty of Pharmaceutical Sciences, Kobe Gakuin University, Kobe 650-8586, Hyogo, Japan; mmtakeda@pharm.kobegakuin.ac.jp; 4 Department of Pharmacy, Faculty of Pharmacy, Mahidol University, Bangkok 10400, Thailand

## Error in Figure

In the original publication [1], there was a mistake in Figure 5B as published. In Figure 5B, both Sox9 and Aggrecan are mentioned in the caption; however, in the published paper, only the Aggrecan figure is shown. The corrected Figure 5B of Sox9 appears below. The authors state that the scientific conclusions are unaffected. This correction was approved by the Academic Editor. The original publication has also been updated.

## Figures and Tables

**Figure 5:**
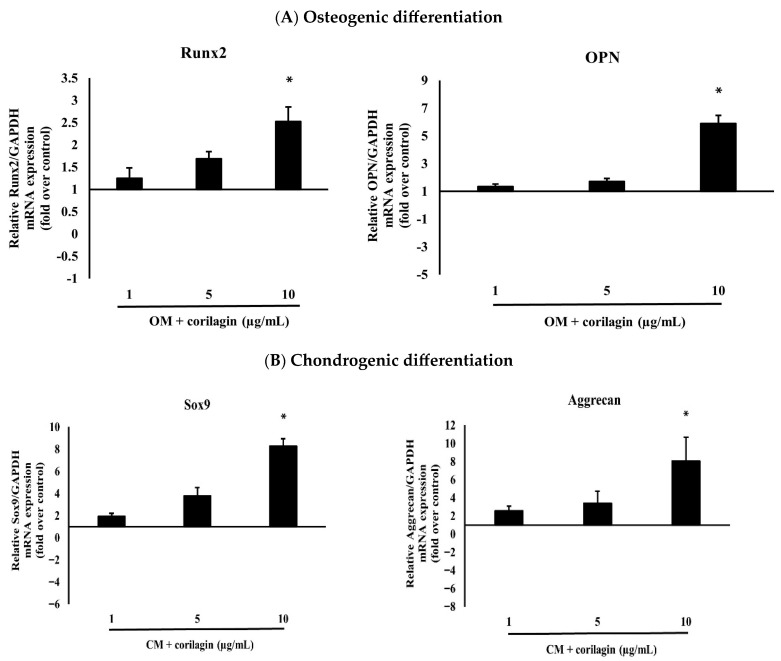
(**A**) The effects of different doses of corilagin (1, 5 and 10 µg/mL) on osteogenic differentiation potentials (Runx2, OPN) of BM-MSCs on day 21. Gene expression of differentiation potentials was assessed using real-time PCR. The bar means SD. Data is shown as mean ± SD. Statistical significance was calculated using a one-way ANOVA test and significance is represented on graphs as * *p*-value < 0.05. (**B**) The effects of different doses of corilagin (1, 5 and 10 µg/mL) on chondrogenic differentiation potentials (Sox9, Aggrecan) of BM-MSCs on day 21. Gene expression of differentiation potentials was assessed using real-time PCR. The bar means SD. Data is shown as mean ± SD. Statistical significance was calculated using a one-way ANOVA test and significance is represented on graphs as * *p*-value < 0.05.
